# The progress of induced pluripotent stem cells derived from pigs: a mini review of recent advances

**DOI:** 10.3389/fcell.2024.1371240

**Published:** 2024-06-24

**Authors:** Jaime A. Neira, J. Vanessa Conrad, Margaret Rusteika, Li-Fang Chu

**Affiliations:** ^1^ Department of Biochemistry and Molecular Biology, Cumming School of Medicine, University of Calgary, Calgary, AB, Canada; ^2^ Faculty of Veterinary Medicine, University of Calgary, Calgary, AB, Canada; ^3^ Reproductive Biology and Regenerative Medicine Research Group, University of Calgary, Calgary, AB, Canada; ^4^ Alberta Children’s Hospital Research Institute, Calgary, AB, Canada; ^5^ Biomedical Engineering Graduate Program, University of Calgary, Calgary, AB, Canada

**Keywords:** porcine pluripotent stem cells, cellular reprogramming, induced pluripotent stem cells, embryonic stem cells, transgene-free

## Abstract

Pigs (*Sus scrofa*) are widely acknowledged as an important large mammalian animal model due to their similarity to human physiology, genetics, and immunology. Leveraging the full potential of this model presents significant opportunities for major advancements in the fields of comparative biology, disease modeling, and regenerative medicine. Thus, the derivation of pluripotent stem cells from this species can offer new tools for disease modeling and serve as a stepping stone to test future autologous or allogeneic cell-based therapies. Over the past few decades, great progress has been made in establishing porcine pluripotent stem cells (pPSCs), including embryonic stem cells (pESCs) derived from pre- and peri-implantation embryos, and porcine induced pluripotent stem cells (piPSCs) using a variety of cellular reprogramming strategies. However, the stabilization of pPSCs was not as straightforward as directly applying the culture conditions developed and optimized for murine or primate PSCs. Therefore, it has historically been challenging to establish stable pPSC lines that could pass stringent pluripotency tests. Here, we review recent advances in the establishment of stable porcine PSCs. We focus on the evolving derivation methods that eventually led to the establishment of pESCs and transgene-free piPSCs, as well as current challenges and opportunities in this rapidly advancing field.

## Introduction

Recent research in both biomedical and veterinary medicine utilizing the pig (*Sus scrofa*) has demonstrated its application as a superb large animal model. Porcine models offer important advantages over other systems, proving more clinically informative compared to smaller murine models while being more practical and accessible than primates (Vodička et al., 2005; Schook et al., 2015; Niemann, 2019; Bertho and Meurens, 2021; Lunney et al., 2021). A well-annotated genome (Groenen et al., 2012; Warr et al., 2020; Pan et al., 2021), combined with advanced gene-editing techniques (Hammer et al., 1985; Luo et al., 2011; Hryhorowicz et al., 2017; Lee et al., 2017; Yan et al., 2018; Li et al., 2018; Perleberg, Kind, and Schnieke, 2018; Yang and Wu, 2018; Maynard et al., 2021; Li et al., 2022), has enabled the proliferation of pig models for disease modeling and comparative studies due to their similarities to humans in anatomical features, physiology, and immunology (Bendixen et al., 2010; Dawson et al., 2013; Lossi et al., 2016; Pabst, 2020; Bertho and Meurens, 2021; Lunney et al., 2021; Li et al., 2022). Thus, swine are positioned as ideal platforms for pre-clinical experimentation (Rouselle et al., 2016; Schomberg et al., 2017; Duran-Struuck, Huang, and Matar, 2019; Henze et al., 2019; Kim et al., 2021; Sper et al., 2022). For example, porcine pluripotent stem cells (pPSCs) or pPSC-derived endothelial cells have already been shown to improve *in vivo* recovery from myocardial infarction (Gu et al., 2012; Li et al., 2013) and promote angiogenesis (Li et al., 2021). The demonstration of this principle using autologous cell transplantation in swine would provide the large-animal, immunosuppression-free validation that is crucial to understanding the clinical potential of these therapeutic approaches (Martínez-Falguera, Iborra-Egea, and Gálvez-Montón, 2021). Similar work has already demonstrated the therapeutic promise of autologous pPSC-derived cell therapies for treating spinal cord injury (Strnadel et al., 2018), using swine as a highly clinically relevant model (Schomberg et al., 2017). Rapid progress in experimental pig-to-human organ xenotransplantation trials is also promising to address the issue of organ shortage and save countless lives (Lu et al., 2020; Porrett et al., 2022; Locke et al., 2023; Loupy et al., 2023; Moazami et al., 2023). Thus, pPSCs derived from early embryos and reprogrammed from somatic cells hold enormous potential in transforming cell therapy and transplantation strategies, while also contributing to a wide range of applications from comparative and developmental biology to agricultural science (Liu et al., 2014; Song et al., 2022; Li et al., 2022; Conrad et al., 2023; Zhu et al., 2023).

## Early challenges in translating mouse and primate PSC derivation methods to pigs

Pluripotent stem cells (PSCs) have long been derived from murine and primate blastocysts (Evans and Kaufman, 1981; Martin, 1981; Thomson et al., 1995, 1996; 1998; Buehr et al., 2008; Li et al., 2008). Because PSCs represent only transient phases of early embryo development, extensive research has focused on the extrinsic (i.e., signaling pathways) and intrinsic factors (i.e., transcription factors) that regulate sustained self-renewal in culture (Chambers and Smith, 2004; Pan and Thomson, 2007; Sasaki et al., 2008; Ying et al., 2008; Hall et al., 2009; Plath and Lowry, 2011; Graf, Casanova, and Cinelli, 2011; Dejosez and Zwaka, 2012; Adachi and Niwa, 2013; Chen et al., 2015). These investigations laid the groundwork for the reprogramming of somatic cells using defined factors to generate induced pluripotent stem cells (iPSCs) from mice and humans, marking an unparalleled breakthrough in regenerative medicine (Takahashi and Yamanaka, 2006; Takahashi et al., 2007; Yu et al., 2007).

Despite efforts spanning more than three decades, challenges have remained in deriving stable pPSCs routinely (Hall, 2013; Zhang et al., 2022). These challenges can be attributed, at least in part, to an incomplete understanding of the species-specific intricacies of early developmental processes in ungulates compared to more well-studied murine species (i.e., mouse and rat) (Perry and Rowlands, 1962; Lamming, 1993). Accordingly, attempts at deriving PSCs from ungulates faced challenges due to the differences in their developmental staging (Evans et al., 1990). For example, blastocysts in ungulates, including cows and pigs, undergo enormous expansion, forming structures such as the embryonic disc, chorion, and allantois, before eventually attaching to the endometrium (Lamming, 1993). Implantation of the ungulate embryo occurs only after a considerable delay, ranging from about 15 days after ovulation in pigs to up to 35 days in cows, compared to only 4 days in mice (Lamming, 1993; Paria, Huet-Hudson, and Dey, 1993). These species-specific differences in morphology and timing during early embryogenesis have influenced efforts to determine how and when pPSCs can be stabilized *in vitro*. Many review articles have elegantly summarized these past efforts in detail (Talbot and Blomberg, 2008; Blomberg and Telugu, 2012; Gandolfi et al., 2012; Malaver-Ortega et al., 2012; Koh and Piedrahita, 2014; Gonçalves, Ambrósio, and Piedrahita, 2014; Ezashi, Yuan, and Roberts, 2016; Han et al., 2019; Zhang et al., 2022), much of which could not be included in this mini review due to limitations in scope.

Recent years have been remarkably productive, and major progress has been made with the generation of stable pPSCs from pre- and peri-implantation embryos (Choi et al., 2019; Gao et al., 2019; Kinoshita et al., 2021; Zhi et al., 2022). Concurrently, the derivation of transgene-free piPSCs using non-integrating cellular reprogramming techniques has finally been reported (Li et al., 2018; Yoshimatsu et al., 2021; Conrad et al., 2023; Zhu et al., 2023). Herein, we review this recent progress and the remaining challenges of this rapidly evolving field.

## Recent progress in pPSCs derived from porcine embryos

The derivation of pESCs from early porcine embryos has been reported since the 1990s. ESC-like cell lines have been derived from embryos ranging between embryonic days 5 (E5) to 11 (E11) post-fertilization, a range which spans most of the pre-implantation, pre-gastrulation developmental period. In particular, these efforts have focused on the inner cell mass (ICM) or the epiblasts of early or hatched blastocysts (∼E5–E8) (Evans et al., 1990; Li et al., 2003; Brevini et al., 2010; Hou et al., 2016; Choi et al., 2019; Zhang et al., 2019; Gao et al., 2019), or the embryonic disc of expanding bilaminar blastocysts (∼E8–E11) (Evans et al., 1990; Strojek et al., 1990; Hochereau-de Reviers and Perreau, 1993; Kinoshita et al., 2021; Zhi et al., 2022). However, complete and conclusive characterizations of most ESC-like lines have not been established. Preliminary characterizations have been consistently performed based on cell and colony morphology and the presence of canonical pluripotency markers, but these results have been remarkably variable between reports. Importantly, the more stringent tests of pluripotency (e.g., teratoma generation, chimeric potential, and germline transmission) remain to be comprehensively demonstrated.

Depending on the embryonic stage of origin, the signaling and culture conditions that allow for a stable expansion of the transient porcine pluripotent cell population can vary significantly. One example is the derivation of expanded potential stem cells (EPSCs) from mice (Yang et al., 2017), humans, and pigs (Gao et al., 2019). Based on combinatory small molecule screens, Gao et al. (2019) described a porcine EPSC (pEPSC) medium, using a cocktail of small molecules including a GSK3 inhibitor (CHIR99021), a SRC inhibitor (WH-4-023), a tankyrase inhibitor (XAV939), vitamin C, LIF, and activin A in an N2B27-based medium (Gao et al., 2019). The pEPSC medium enabled the derivation of stable pEPSC lines from pre-implantation blastocysts (day 5, *in vivo* derived; or day 7, parthenogenetically derived). The EPSCs could be maintained over 40 passages on STO feeders with an undifferentiated morphology and a normal karyotype. This study also concluded that pEPSCs have the potential to contribute to both embryonic and extraembryonic trophoblast lineages in chimeric assays. Future research is still required to better define the properties of the “expanded potential” state in relation to totipotency (Posfai et al., 2021).

Using a similar rationale to optimize derivation conditions, Choi et al. (2019) developed a pig ESC medium that contains KnockOut Serum Replacement (KOSR), lipid concentrate, FGF2, activin A, and WNT signaling modulators (CHIR99021 and IWR-1). This medium not only allowed for the expansion of SOX2-expressing cells from the ICM outgrowths, but also enabled the derivation of stable pESC lines from both IVF- and parthenogenetically-derived embryos (Choi et al., 2019). pESC lines were stably maintained for more than 1 year while maintaining stemness and a normal karyotype (Choi, Lee, Oh, Kim, Lee, Woo, et al., 2020). Interestingly, RNA-seq analysis showed that pESCs are transcriptionally closer to an epiblast-like state than to the ICM state (Secher et al., 2017; Choi, Lee, Oh, Kim, Lee, Kim, et al., 2020).

By carefully isolating the epithelial embryonic disc layer from pig embryonic day 11 pre-gastrulation spherical blastocysts, Kinoshita et al. (2021) derived stable embryonic disc stem cells (EDSCs) using an “AFX” medium (referred to as pEDSC medium hereafter: an N2B27-based medium supplemented with activin A, FGF2, and XAV939), and maintained the cells under hypoxic conditions (5% O_2_) at 38.5°C. Remarkably, the pEDSCs were able to readily adapt to feeder-free environments on fibronectin and laminin matrices. This represents a step forward in the complete and defined characterization of PSC maintenance, as feeder cells often suffer from batch-to-batch variabilities and could interfere with downstream analysis (Heng et al., 2004; Mallon et al., 2006). Transcriptomic analyses indicated that pEDSCs are similar to pESCs but distinct from pEPSCs. Interestingly, the pEDSC medium also stabilized EDSCs derived from sheep and bovine embryos, suggesting this may be a common state that can be stabilized across ungulates (Kinoshita et al., 2021).

By tracing the lineage trajectories of the pluripotent epiblast cells from E0–E14 pig pre-implantation embryos using single-cell RNA-seq (scRNA-seq), Zhi et al. (2022) derived stable pig pre-gastrulation epiblast stem cells (pgEpiSCs) from E10 epiblast. The pgEpiSCs could be expanded in a “3i/LAF” medium for more than 240 passages while still retaining the ability to self-renew and differentiate. The 3i/LAF medium shares similarities with some of the pEPSC, pESC and pEDSC counterparts, using a N2B27-based medium, KOSR, CHIR99021, IWR-1, WH-4-023, LIF, activin A, and FGF2. Interestingly, when subjected to chimeric assays, pgEpiSCs only had a limited ability to contribute to the development of the host embryo. RNA-seq analysis showed that transcriptomic differences exist between pEPSCs, pEDSCs and pESCs (Kinoshita et al., 2021). Future research is required to elucidate whether these differences reflect biologically distinct stages of pluripotency or are based primarily on adaptation to the various culture conditions.

## Technical challenges in the derivation of transgene-free piPSCs

**FIGURE 1:**
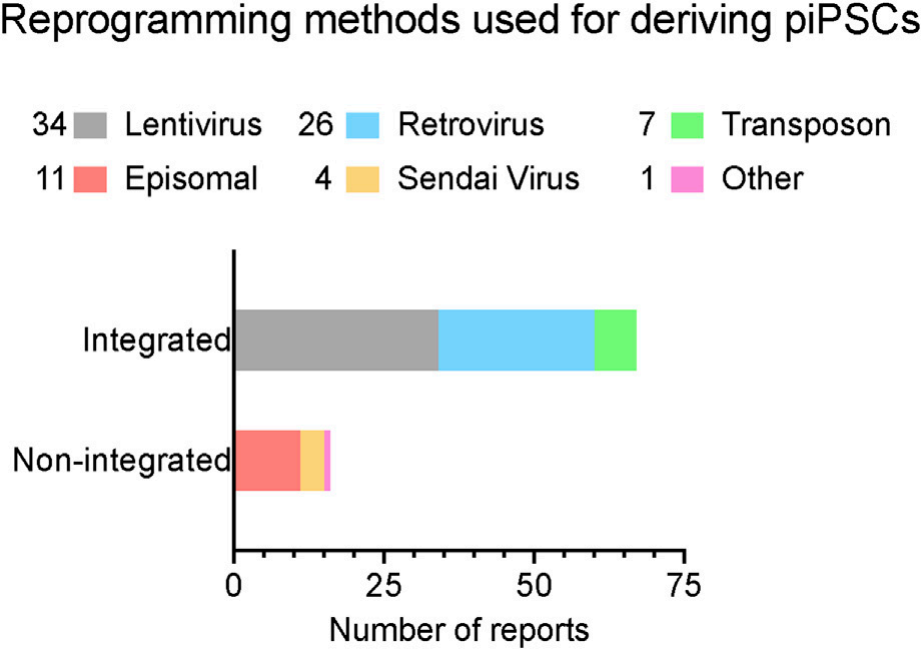
A summary of reported reprogramming methods used in published studies for deriving piPSCs. Reprogramming methods sorted by integration strategy. X-axis lists the number of reports that have been published using each strategy. Number of reports per method noted in legend. Further details are listed in Table 1. Reports were collected by performing systematic searches between September 2023 and January 2024 on NCBI PubMed with the key words “porcine induced pluripotent stem cell” and “piPSC” and “reprogramming”.

The establishment of piPSCs using the Yamanaka reprogramming factors OCT4, SOX2, KLF4, and c-MYC (OSKM) delivered by retroviral/lentiviral vectors was reported in pigs since shortly after the first reported generation of mouse iPSCs, as we have summarized in Figure 1 and comprehensively annotated details in Table 1 (Wu et al., 2009; Esteban et al., 2009; Ezashi et al., 2009). These cell lines displayed conventional PSC properties and could be differentiated into three germ layers *in vitro* and form teratomas. Integrative reprogramming strategies have proven effective for efficiently making piPSC-like colonies from porcine somatic cells and have been used for many applications related to xenotransplantation and immunogenicity (Park et al., 2013; Liu et al., 2013), understanding key developmental signaling (Arai et al., 2013; Xu et al., 2020; Yang et al., 2022; Yuan et al., 2019), and deriving disease-relevant cell types (Gu et al., 2012; Aravalli, Cressman, and Steer, 2012; Yang et al., 2013; Park et al., 2016; Liao et al., 2018; Yu et al., 2022; Liao et al., 2023) (Table 1). However, an inevitable drawback of using integrating methods for introducing reprogramming factors is that they compromise the integrity of the host cell genome, raising their oncogenic potential (Prigione et al., 2011; Chen et al., 2014) and limiting their translational applications (Fan et al., 2013; Kang et al., 2015). There also tends to be an inverse relationship between the integration of a transgene and the expression of its endogenous counterpart (Hall et al., 2012). It is possible that transgene integration may counteract the activation of endogenous pluripotency factors by creating a reliance on the transgene and bypassing the process of complete epigenetic reprogramming, resulting in an unstable and artificial state of pluripotency (Hussein et al., 2014; Du et al., 2015). Thus, the ideal system is one in which piPSCs are transgene- and integration free, making them more faithful and self-sustaining models of pESC-like pluripotency.

**TABLE 1:** Systematic annotation of reprogramming methods used for generating PiPSCs.

**Strategy**	**Reprogramming method**	**Starting cell type**	**Reprogramming Factors**	**Species of the reprogramming factors**	**Teratoma assay**	**Chimeric assays**	**Transgene Free**	**Media Supplementation**	**Reference**
Integrated	Lentivirus	Bone Marrow Cells	OSKM, NANOG, LIN28	Human	Y	N	N	KOSR	Wu et al. (2009)
		Fibroblasts	OSKM	Human	Y	N	N	KOSR + FGF2	Ezashi et al. (2009, 2011)
			OSKM, NANOG, LIN28	Human	Y	N	N	KOSR	Wu et al. (2009)
		Mesenchymal Stem Cells	OSKM, NANOG, LIN28	Human	N	**Y**	N	mTeSR1	West et al. (2010, 2011)
		Adipose Stromal Cells	OSKM	Human	Y	N	N	FBS + bFGF	Gu et al. (2012)
		Fibroblasts	OSKM	Human	N	N	N	N2B27 + BSA + mLIF + PD0325901 + CHIR + PD173074	Rodríguez et al. (2012)
			OSKM, NANOGP8	Human	N	N	N	KOSR + bFGF	(Vanessa J. Hall et al. 2012)
			OSKM, NANOG, LIN28	Human	N	N	N	mTeSR1	Yang et al. (2013), Gallegos-Cárdenas et al. (2015)
								mTeSR1, KOSR + bFGF	Liu et al. (2013)
					Y	N	N	FBS + KOSR + LIF + bFGF	Kwon et al. (2013)
		Adipose-Derived Stem Cells	OSKM	Human	Y	N	N	KOSR + N2B27 + hLIF + PD0325901 + CHIR99021	Zhang et al. (2014)
		Fibroblasts	OSKM	Human	Y	N	N	FBS	Liao (2014)
								FBS + KOSR + mLIF + bFGF	Ao et al. (2014)
					N	N	N	LIF + CHIR99021 + PD0325901; bFGF	Choi et al. (2016)
				Pig	Y	Chimeric embryos	N	PD0325901 + CHIR99021 + LIF; FGF2	Secher et al. (2017)
			OSKM, NANOG, LIN28	Human	N	N	N	KOSR, FBS, LIF	Kwon D. J. et al. (2017)
				Mouse	Y	Chimeric embryos	N	LIF + FGF2 + PD0325901 + CHIR99021 + thiazovivin + GFx	Fukuda et al. (2017)
			OSKM	Human	N	N	N	PL + BMP4 + SCF + IL-6 + CHIR99021 + SB431542 + PD0325901	Ma et al. (2018)
								FBS	Liao et al. (2018)
								FBS + hLIF + bFGF + CHIR99021 + SB431542	Shen et al. (2019)
								N2B27 + hLIF + Vc + ITS-A + PD0325901 + CHIR99021 + Gö6983	Habekost et al. (2019)
			OSKM, NANOG, LIN28	Human	Y	N	N	mTeSR1	Burrell et al. (2019)
			OSKM	Human	N	N	N	KOSR + bFGF	Machado et al. (2020)
								N2B27 + KOSR + CHIR99021 + PD0325901 + hLIF + pLIF	Shi et al. (2020)
								Not specified	Jiang et al. (2022)
				Mouse	N	N	N	KOSR + LIF; KOSR + bFGF; KOSR +bFGF + LIF	Pieri et al. (2022)
		Fibroblasts; Sertoli Cells	OSKM	Pig	N	N	N	FBS + LIF + bFGF + CHIR99021 + SB431542	Yu et al. (2022)
		Urine-derived Cells	OSKM	Mouse	N	N	N	KOSR + bFGF	Recchia et al. (2022)
		Fibroblasts	OSKM	Not specified	N	N	N	KOSR + FGF2	Guo et al. (2023)
				Mouse	N	N	N	KOSR + N2B27 + BSA + bFGF + LIF + CHIR99021 + PD0325901 + SB431542 + Vc	Zhou et al. (2023)
				Human	N	N	N	FBS + KOSR + LIF	Baek et al. (2023)
			OSKM, NANOG, LIN28	Human	N	N	N	FBS + KOSR + LIF	Baek et al. (2023)
			OSKM, BRG1	Human	N	N	N	FBS + bFGF + hLIF + dorsomorhpin	Ren et al. (2024)
			OSKM, TBX3	Human, Pig	Y	Chimeric embryos	N	FBS + LIF + bFGF + CHIR99021 + SB431542	Shen et al. (2024)
Integrated	Retrovirus	Fibroblasts	OSKM	Human	Y	N	N	FBS + bFGF	Esteban et al. (2009)
				Mouse	Y	N	N	FBS + bFGF	Esteban et al. (2009)
				Human	Y	N	N	FBS + KOSR + LIF + bFGF	Ruan et al. (2011)
				Mouse	N	N	N	KOSR + hLIF	Thomson et al. (2012)
					Y	Chimeric blastocysts	N	FBS + LIF + bFGF	Cheng et al. (2012)
			SKM	Mouse	Y	N	N	FBS + KOSR + LIF + bFGF	Montserrat et al. (2012)
		Mesenchymal Stem Cells	OK	Pig	Y	N	N	KOSR or FBS + hLIF	Liu et al. (2012)
		Fibroblasts	M, NR5A2	Mouse	N	N	N	KOSR + N2B27 + BSA + hLIF + bFGF	Wang et al. (2013)
			NR5A2	Mouse	N	N	N	KOSR + N2B27 + BSA + hLIF + bFGF	Wang et al. (2013)
			OSKM	Human	N	N	N	FBS + KOSR + bFGF	Park et al. (2013)
						Chimeric embryos	N	KOSR + forskolin + pLIF	Fujishiro et al. (2013)
								KOSR + forskolin + pLIF + PD0325901 + CHIR99021	Arai et al. (2013)
				Mouse	Y	N	N	KOSR + N2B27 + BSA + hLIF + bFGF	Wang et al. (2013), Wei et al. (2020), Li et al. (2021)
			OSKM, NR5A2, TBX3	Mouse	Y	N	N	KOSR + N2B27 + BSA + hLIF + bFGF	Wang et al. (2013)
			OSKM	Human	Y	N	N	KOSR + bFGF	Li et al. (2014)
				Mouse	Y	N	N	KOSR + N2B27 + LIF + bFGF + PD0325901 + CHIR99021 + SB431542 + Vc	Gu et al. (2014)
			OSK, miR302a, miR302b, miR200c	Mouse	Y	N	N	KOSR + LIF	Ma et al. (2014)
			OSKM, TERT	Human	Y	Chimeric blastocysts	N	FBS + KOSR + LIF + bFGF	Gao et al. (2014)
			OSKM	Human	Y	N	N	FBS + hLIF + FGF2 + BMP4 + CHIR99021 + SB431542	Zhang et al. (2015)
					N	Chimeric blastocysts	N	FBS + bFGF + SCF	Park et al. (2016)
						N	N	FBS + hLIF + bFGF + CHIR99021 + SB431542	Yu et al. (2017)
			OSKM, TET1, KDM3A	Human, Mouse	Y	N	N	KOSR + FBS + hLIF + HDACi	Mao et al. (2017)
			OSKM	Pig	Y	Chimeric embryos	N	FBS + hLIF + CHIR99021 + PD0325901 + AlbuMAX	Zhang et al. (2018)
			OSKM, ESRRB	Human, Pig	N	N	N	FBS + hLIF + FGF2 + BMP4 + CHIR99021 + SB431542	Yang et al. (2018)
		Fibroblasts; Pericytes	OSKM	Pig	N	Chimeric embryos	N	N2B27 + KOSR + LIF + CHIR99021 + (S)-(+)-Dimethindene Maleate + Minocycline Hydrochloride + Vc	Xu et al. (2019)
		Sertoli Cells	OSKM	Human	Y	N	N	KOSR + FBS + bFGF + hLIF	Setthawong et al. (2019)
		Fibroblasts	OSKM	Human	N	N	N	KOSR + FBS + bFGF + hLIF	Setthawong et al. (2021)
			OSKM, LIN28	Human	Y	N	N	KOSR + FBS + mLIF + bFGF	Chakritbudsabong et al. (2021)
Integrated	Transposon (Sleeping Beauty)	Fibroblasts	OSKM	Mouse	Y	N	N	KOSR + bFGF	Kues et al. (2013)
			OSKM, NANOG, LIN28	Human, Pig	N	N	N	KOSR + mLIF + bFGF	Petkov et al. (2013)
								FBS + KOSR + mLIF	Petkov et al. (2013)
								FBS + SAHA + VPA + NaB + Vc	Petkov et al. (2016)
	Transposon (PiggyBac)	Fibroblasts	OSKM	Human	N	N	N	N2B27 + CHIR99021 + PD0325901 + hLIF	Yu et al. (2018)
			OSKM, NANOG, LIN28, LRH1, RARG	Human, Pig	N	Chimeric embryos	N	N2B27 + CHIR99021 + WH-4-023 + XAV939/IWR1 + Vc + LIF + Activin + FBS	Gao et al. (2019)
				Cow, Human	N	N	N	N2B27 + CHIR99021 + WH-4-023 + XAV939 + Vc + LIF + Activin + FBS	Zhou et al. (2023)
				Pig, Human	N	Chimeric embryos	N	N2B27 + CHIR99021 + WH-4-023 + XAV939 + Vc + LIF + Activin + FBS	Zhou et al. (2023)
Non-integrated	Germinal Vesicle Oocyte Extract	Fibroblasts	N/A	Pig	Y	Chimeric blastocysts	N/A	ES medium	Bui et al. (2012)
	Sendai Virus	Fibroblasts	OSKM	Human	N	N	N	Not specified	Juhasova et al. (2015)
					Y	N	N	FBS + KOSR + bFGF	Congras et al. (2016)
								KOSR + bFGF	Strnadel et al. (2018)
					N	N	Y	KOSR + bFGF	Baek et al. (2023)
	Episomal Plasmid	Fibroblasts	OSKM, NANOG, LIN28	Human, Mouse	Y	N	N	KOSR + PD0325901 + CHIR99021 + hLIF + VPA	Telugu et al. (2010)
			OSKM	Mouse	Y	N	N	FBS + KOSR + LIF + bFGF	Montserrat et al. (2011)
			OSK	Human	N	N	N	KOSR	Aravalli et al. (2012)
			OSKM	Mouse	N	N	N	FBS + SCF + bFGF	Park et al. (2013)
			OSKM, NANOG, LIN28, NR5A2, miR302/367	Human	Y	Chimeric blastocysts	N	N2B27 + CHIR99021 + PD0325901 + mLIF	Du et al. (2015)
			OSKM, LIN28, shP53	Human	Y	N	Y	KOSR + bFGF + PD0325901 + CHIR99021	Li et al. (2018)
							N	KOSR + hLIF + hFGF2 + BIRB796 + SP600125 + LDN193189 + CHIR99021 + PD0325901 + SB431542	Yuan et al. (2019)
			OSKM, NANOG, LIN28, KLF2, KDM4D, GLIS1, mP53DD	Human, Mouse, Marmoset	N	N	Y	KOSR + bFGF + Activin A + TGFb1 + IWP2	Yoshimatsu et al. (2021)
			OSKM, NANOG, LIN28, NR5A2, BAF60A, miR302-367	Human	Y	N	N	E8 + Activin A + CHIR99021 + IWR1 + LIF	Jiao et al. (2022)
			OSKM, BCL2L1	Human	Y	N	Y	N2B27 + KOSR + Vc + bFGF + Activin A + hLIF + CHIR99021 + IWR1 + WH-4-023	Zhu et al. (2023)
			OSKM, NANOG, LIN28, SV40LT, miR302-367	Human	Y	N	Y	KOSR + FGF2 + Activin A + CHIR99021 + IWR1	Conrad et al. (2023)

Various delivery methods are highlighted by color. OSKM = OCT3/4, SOX2, KLF4, MYC. Y, yes; N, No; KOSR, KnockOut™ Serum Replacement; FBS, Fetal Bovine Serum; BSA, bovine serum albumin.

Attempts to make transgene free piPSCs using episomal reprogramming methods continued for years, but the challenges of integration and retention stubbornly persisted (Telugu, Ezashi, and Roberts, 2010; Montserrat et al., 2011; Aravalli, Cressman, and Steer, 2012; Park et al., 2013). Although piPSC-like cultures were produced, none of the resulting cell lines were able to demonstrate complete transgene loss (Du et al., 2015). Even in the case of Sendai virus-based reprogramming, which uses minus-strand RNA as a template to encode reprogramming factors and is thus incapable of integrating into the host genome, the viral sequences were either maintained in the derived piPSC populations (Congras et al., 2016) or not shown to be absent in the pluripotent state (Juhasova et al., 2015; Strnadel et al., 2018). The exact causes of transgene retention are unclear, but issues with cell viability, proliferative advantage, and incomplete signaling conditions are all potential factors (Silva et al., 2008; Golipour et al., 2012; Chia et al., 2017). For example, it is possible that cells which retain the transgenes gain a competitive advantage over cells that do not, as the early reprogramming process is known to result in a significant increase in cell cycling and mitotic rate (Ruiz et al., 2011; Guo et al., 2014). Due to these challenges, the establishment of genuine transgene-free piPSCs remained elusive.

## Recent progress in transgene-free piPSC derivation

More recently, using eight episomal plasmids encoding a set of eleven reprogramming factors, Yoshimatsu et al. (2021) were able to carefully study the reprogramming intermediates and show that somatic cells temporarily acquired a neural stem cell-like state during the transition. Stable piPSC colonies were established in the process and expanded in an “ESM” medium, which includes activin A, TGF1, and IWP2 (a WNT signaling inhibitor), similar to the conditions described above for deriving pESCs and ESC-like cells. In this reprogramming regime, the piPSCs lost the transgenes in approximately five passages after clonal isolation and expansion. Interestingly, this reprogramming protocol was also applied to the establishment of transgene-free marmoset and dog iPSCs, highlighting potential shared reprogramming paradigms and mechanisms.

Building on the successful establishment of pgEpiSCs (Zhi et al., 2022), Zhu et al. (2023) reprogrammed fibroblasts by electroporating up to six episomal plasmids encoding seven reprogramming factors to establish episomally derived piPSCs (epi-iPSC). These epi-iPSCs were maintained in the aforementioned 3i/LAF medium, lost their episomal plasmids around passage 8, and are remarkably similar to pgEpiSCs in their transcriptomic signatures, proliferation profile and capacity for self-renewal.

Similarly, using the pESC medium reported by Choi et al. (2019), Conrad et al. (2023) established transgene-free piPSCs using three episomal plasmids encoding seven reprogramming factors. As had been reported in human iPSCs, co-electroporating a microRNA302/367 cassette greatly enhanced the efficiency of primary colony formation (Kuo et al., 2012; Howden et al., 2015). The clonally amplified piPSC lines lost detectable episomal plasmids by around passage 10 and maintained their undifferentiated morphology for more than 50 passages in the pESC medium. These transgene-free piPSCs were very similar to pESCs in gene expression signatures and were capable of differentiating into progenitors representing the primary three germ layers and forming teratomas in immunocompromised mice. Compellingly, when used to model the segmentation clock, these piPSCs preserve an ungulate-specific developmental allochronic phenotype *in vitro* (Conrad et al., 2023; Lázaro et al., 2023).

Across these reports (Table 1, orange colored section), culture conditions shared certain key commonalities, including the use of serum replacement and bFGF. However, the lack of consistency in many other components (such as TGF- and WNT-modulators) points to at least two possibilites; these cell lines may represent meaningfully divergent pluripotency states with distinct signalling requirements, or some of these components may not be essential for maintaining porcine pluripotency. Further research will be necessary to elucidate these differences.

## Current challenges and future directions of pPSC research

### Demonstration of complete developmental potential

Our understanding of pluripotency remains incomplete. Since cellular reprogramming is known to be stochastic and highly variable, a state of complete, genome-wide reprogramming (absent of somatic imprinting or methylation patterns) needs to be clearly demonstrated. To validate complete reprogramming of the produced iPSC lines, the generation of an all-iPSC animal is ultimately required (Nagy et al., 1993; Tam and Rossant, 2003), a feat thus far only achieved by high quality mouse iPSCs (Zhao et al., 2009; Kang et al., 2009; Boland et al., 2009). Similarly, germline competence has only been conclusively shown for mouse and rat iPSCs (Okita et al., 2007; Hamanaka et al., 2011). Despite the recent advancements in pPSC research, it remains to be determined whether any of the pEPSCs, pEDSCs, pESCs, pgEpiSCs, or transgene-free piPSCs are germline competent and whether they could contribute to the development of all-PSC animal (West et al., 2011; Secher et al., 2015; Wang et al., 2016; Posfai et al., 2021). It will also be beneficial to compare existing piPSC and pPSC derivation methods more systematically, to establish efficient and reproducible protocols that can be scaled and adopted more widely.

### Improved understanding of the porcine pluripotent state

A variety of states of pluripotency have been characterized by adapting novel cell culture conditions. For example, these include naïve (Ying et al., 2008; Nichols et al., 2009; Nichols and Smith, 2009), primed (Brons et al., 2007; Tesar et al., 2007), region-selective (Wu et al., 2015), rosette-stage (Neagu et al., 2020), intermediate (Zhang et al., 2015; Yu, Wei, Sun, et al., 2021), and formative (Smith, 2017; Zhi et al., 2022) states, which represent a diverse spectrum of states from early mammalian embryos (Hall and Hyttel, 2014; Bernardo et al., 2018). To pinpoint the exact state of reprogrammed piPSC lines, it is necessary to compare with embryos or embryo-derived PSCs as the “gold standard” (Weefrnig et al., 2007; Chung et al., 2014; Yang et al., 2018; Jiang et al., 2022; Conrad et al., 2023). Systematic, robust, cross-species comparative studies will continue to be highly informative to understanding these cell types in relation to each other (Habekost et al., 2019; Simpson et al., 2023), and would in turn provide insights into the conserved mechanisms of early mammalian development (Ben-Nun et al., 2011; Shahbazi et al., 2017; Boroviak and Nichols, 2017; Yu, Wei, Sun, et al., 2021; Soto et al., 2021; Zywitza et al., 2022; Déjosez et al., 2023; MacCarthy et al., 2024). The continued development of PSCs from new species will be instrumental to this understanding (Rayon et al., 2020; Lázaro et al., 2023). A promising development is the generation of a chimeric factor, SOX2-17, or super-SOX, which greatly enhanced the derivation of iPSCs from pigs as well as mice, humans, cynomolgus macaques, and cows (MacCarthy et al., 2024). The SOX2-17 factor stabilized SOX2/OCT4 dimerization and improved the ability to form all iPSC-mice by tetraploid complementation. This factor also supported a naïve reset in multiple species, suggestive of a conserved mechanism that could be further applied to many other species.

### Applied differentiation of pPSCs to functional cell types

Finally, the direct differentiation of pPSCs into functional, mature cell types for regenerative medicine applications remains to be fully investigated. While early works have shown that pPSCs can readily differentiate into lineage-specific progenitors using protocols already developed for murine and human PSCs, tailoring the differentiation paradigm specifically for producing mature porcine cells will ultimately be required (Gu et al., 2012; Aravalli, Cressman, and Steer, 2012; Yang et al., 2013; Liao et al., 2018; Jeon et al., 2021). Nevertheless, progress is rapidly unfolding. A recent study showed that pgEpiSCs can be differentiated into skeletal muscle fibers and form three-dimensional meat-like tissues (Zhu et al., 2023). When combined with other improvements in the expansion of primary muscle stem cells and adipose-derived stem cells, these represent a step forward to the development of cultured meat products from an unlimited cellular source (Li et al., 2022; Song et al., 2022). Consistent developments in xenotransplantation are equally promising (Strnadel et al., 2018; Porrett et al., 2022; Locke et al., 2023; Loupy et al., 2023; Moazami et al., 2023; Wang et al., 2023), with pPSCs providing the ideal platform for generating pigs that can be readily modified and adapted according to clinical need. For example, the knockout of key immunogenic antigens has been proven to increase immune tolerance in pig-to-human xenotransplantation (Liu et al., 2013; Xu et al., 2022). Recent advances in whole- or partial-embryo modelling could also unlock new, previously inaccessible stages of developmental biology once they are translated to swine (Liu et al., 2021; Yu, Wei, Duan, et al., 2021; Tarazi et al., 2022; Weatherbee et al., 2023; Amadei et al., 2022; Liu et al., 2023; Oldak et al., 2023; Wu et al., 2023). Broadly speaking, the field is at an exciting juncture, with the potential for groundbreaking developments in regenerative medicine, disease modeling, and cell therapy.
